# Impact of extracellular matrix derived from osteoarthritis subchondral bone osteoblasts on osteocytes: role of integrinβ1 and focal adhesion kinase signaling cues

**DOI:** 10.1186/ar4333

**Published:** 2013-10-09

**Authors:** Indira Prasadam, Saba Farnaghi, Jian Q Feng, Wenyi Gu, Samuel Perry, Ross Crawford, Yin Xiao

**Affiliations:** 1 Institute of Health and Biomedical Innovation, Queensland University of Technology, Kelvin Grove Campus, Brisbane, Queensland 4059, Australia; 2 Prince Charles Hospital, Brisbane, Queensland, Australia; 3 Texas A&M Health Science Center, H, Round Rock, TX, USA

## Abstract

**Introduction:**

Our recent study indicated that subchondral bone pathogenesis in osteoarthritis (OA) is associated with osteocyte morphology and phenotypic abnormalities. However, the mechanism underlying this abnormality needs to be identified. In this study we investigated the effect of extracellular matrix (ECM) produced from normal and OA bone on osteocytic cells function.

**Methods:**

De-cellularized matrices, resembling the bone provisional ECM secreted from primary human subchondral bone osteoblasts (SBOs) of normal and OA patients were used as a model to study the effect on osteocytic cells. Osteocytic cells (MLOY4 osteocyte cell line) cultured on normal and OA derived ECMs were analyzed by confocal microscopy, scanning electron microscopy (SEM), cell attachment assays, zymography, apoptosis assays, qRT-PCR and western blotting. The role of integrinβ1 and focal adhesion kinase (FAK) signaling pathways during these interactions were monitored using appropriate blocking antibodies.

**Results:**

The ECM produced by OA SBOs contained less mineral content, showed altered organization of matrix proteins and matrix structure compared with the matrices produced by normal SBOs. Culture of osteocytic cells on these defective OA ECM resulted in a decrease of integrinβ1 expression and the de-activation of FAK cell signaling pathway, which subsequently affected the initial osteocytic cell’s attachment and functions including morphological abnormalities of cytoskeletal structures, focal adhesions, increased apoptosis, altered osteocyte specific gene expression and increased Matrix metalloproteinases (MMP-2) and -9 expression.

**Conclusion:**

This study provides new insights in understanding how altered OA bone matrix can lead to the abnormal osteocyte phenotypic changes, which is typical in OA pathogenesis.

## Introduction

Bone matrix serves as an organized framework for bone as a tissue, offering mechanical support and mediating biological activities of bone cells and signals that maintain bone homeostasis and remodelling [1]. Bone cells, like most other matrix-associated cells, cannot survive or differentiate without adhesion to their matrix [2,3]. Consequently, bone cell morphology and functions can depend strongly on matrix quality under conditions in which biological signals are constant. In osteoarthritis (OA) it is well-known that subchondral bone matrix, structure, organisation, composition and mineralisation are abnormal when compared to normal bone [4].

Osteocytes are the most abundant and longest-living cells in the adult skeleton. The importance of osteocytes in regulating bone remodeling and turnover has been generally acknowledged [5]. Our recent study demonstrated that various functional and morphological properties of osteocytes appear to be hampered in patients with OA, indicating that these cells could play an important pathological role in subchondral bone sclerosis [6]. However, the potential molecular mechanism behind this abnormal osteocyte behaviour in OA patients is yet to be identified.

*In vivo,* osteocyte cells under normal conditions, contact a complex mixture of secreted ‘extracellular matrix’ (ECM) proteins called the bone matrix. The bone matrix isolates osteocytes from each other and instead osteocytes interact with other osteocytes and other bone cells by an elaborate network of osteocytes (dendritic) processes. The contact with the bone matrix is a critical mechanism providing cues *via* cytoplasmic processes called canalicules to form a cellular network to sense efficiently both mechanical and systemic stimuli [7]. On the other hand, it seems that osteocytes which become transformed in diseases such as osteoporosis and OA are characterised by loose contact with ECM substrate leading to morphological and functional bony changes [6,8]. Primarily based on our previous observations, in this study we hypothesised that altered mineralisation and the ECM quality of the subchondral bone matrix is the trigger for the osteocyte abnormalities seen in OA.

*In vivo* cell adhesion to the ECM is mediated by integrinβ1 receptors. Bone ECMs are composed of several macromolecules including fibronectin, laminin, collagens and proteoglycans. A number of these ECM proteins contain the three amino acid sequence Arg-Gly-Asp (RGD), which is exclusively recognised by corresponding integrinβ1 receptors [9,10]. Attachment of integrins with the above macromolecules can activate the downstream signalling focal adhesion kinase (FAK) and vinculin that can initiate a cascade of phosphorylation events that fine-tune cell-type-specific phenotypes [11]. Maintenance of integrin linkages is essential for cell adhesion, proper cytoskeletal organisation and function of the specific cell types. It has been demonstrated previously that disruption of these attachments, *via* addition of neutralising antibodies or peptides, can induce cells to detach from the ECM resulting in apoptosis, structural alterations and cellular dysfunction. The aim of this study is to test how normal and OA bone ECM differentially regulates the function of the osteocytes. The other objective of the present study is to reveal the critical role of cell-matrix adhesions governing this process, notably involving the integrinβ1-FAK signalling axis.

## Methods

### Subchondral bone osteoblast (SBO) isolation and characterisation

The Ethics Committee of Queensland University of Technology and the Prince Charles Hospital approved this study and the participants’ written consent was obtained according to the Declaration of Helsinki (Ethics Number: 0700000157). Knee bone specimens were taken within 5 mm of the subchondral bone plate as described previously in our studies [12-14]. OA SBOs were cultured from bone sourced from the medial compartment of the knee from patients suffering advanced OA, where the cartilage was degraded and showed prominent subchondral bone sclerosis and density (n = 5) (age: 61.5 ± 4 years). Normal SBOs were cultured from bone collected from trauma patients who were undergoing above the knee amputations with no evidence of subchondral bone sclerosis or cartilage degeneration on top of it (n = 4) (age: 60.1 ± 6 years). The criteria for the OA diagnosis were those established according to the American College of Rheumatology [15]. None of the normal patients had any musculoskeletal disorders, such as osteoporosis. SBOs were isolated according to the method described by Beresford [16,17]. Isolated normal and OA SBOs were characterised for their phenotype as described in our previous studies [12,13]. Passage one SBOs were used for this study.

### Subchondral bone osteoblast differentiation and preparation of de-cellularised matrices

Osteogenic differentiation of normal and OA SBOs (20,000 cells per well) was performed in (D)MEM medium containing osteogenic supplements (10 nM dexamethasone, 10 mM β-glycero-phosphate, 50 μg/mL ascorbic acid) on coverslips (NUNC, Roskilde, Denmark) (placed on 24 well plates) for five weeks. After five weeks, SBOs were rinsed two times in 1X PBS. Next, to produce the de-cellularised matrices, 0.02 M ammonium hydroxide in ddH_2_O was applied for 20 to 30 minutes at room temperature intermittently visualising under a light microscope for cell roundup and lysis. Then ammonium hydroxide was removed by inverting the culture surface, with plates washed several times with sterile 1X PBS before seeding osteocytes on top of them.

### Characterisation of de-cellularised matrices

The removal of cells and the retention of a collagenous matrix were verified following de-cellularisation. Briefly, cells were fixed in 4% paraformaldehyde for ten minutes, washed three times with 1X PBS and permeabilised with 0.1% Triton for five minutes and stained with ProLong Gold Antifade Reagent (Invitrogen, Life Technologies Australia Pty Ltd, Victoria, Australia) with 4',6-diamidino-2-phenylindole (DAPI) on a glass slide. The absence of nuclei was confirmed using fluorescence microscopy (Zeiss, using AxioVision Image analysis software). The presence of collagen I and various ECM proteins in the de-cellularised matrices was confirmed by immunostaining and western blotting following the protocol described in our previous studies [12,13]. The presence of mineral content was confirmed by staining matrices with 1% alizarin red. Scanning electron microscopy (SEM) with energy dispersive X-ray analysis (SEM/EDX) (FEI Quanta 200 Environmental SEM equipped with an Evarhart Thomley secondary electron detector) was used to examine the morphology of the calcium phosphate deposit and to obtain the elemental composition in normal and OA matrices.

### MLOY4 osteocyte cell line culture

The well-characterised osteocytic line, MLOY4 (provided by Dr. Lynda Bonewald) [18] was cultured on T75 tissue culture flasks coated with type I collagen (0.30 mg/ml; Sigma, St. Louis, MO, USA) in osteocyte culture medium containing alpha modified essential medium (αMEM; GIBCO BRL, Grand Island, NY, USA), 5% fetal bovine serum (FBS; Hyclone, Logan, UT, USA), 5% calf serum (CS), and 1% penicillin and streptomycin (GIBCO BRL).

### Seeding MLOY4 cells on normal and osteoarthritis matrices

MLOY4 cells (10,000 cells/cover slip) were seeded directly on the top of the normal and OA matrices prepared above and incubated in osteocyte medium at 37°C containing 5% CO_2_/95% atmospheric air at different time points.

### Integrin and focal adhesion kinase signalling studies

To confirm the integrinβ1 mediated molecular mechanism, MLOY4 cells were cultured on normal and OA matrices in the presence or absence of blocking-integrinβ1 (10 μm) antibody (Clone P5D2, Chemicon, Millipore, MERCK Pvt Ltd, Victoria, Australia) or with an irrelevant antibody. P5D2 react with the human *β*_1_ integrin subunit and block adhesion of cells to ECM proteins. FAK activation and de-activation was analysed by anti-phospho- FAK and anti-total FAK antibodies (Cell Signaling Technology, Gene search Pty Ltd, Queensland, Australia).

### Scanning electron microscopy

The morphology of osteocytes grown on normal and OA osteoblast matrices were assessed by SEM as described previously [19]. (+/- MLOY4 cells) were fixed with a solution containing 3% (v/v) glutaraldehyde in 0.1 M sodium cacodylate buffer solution (pH 7.3) for one hour at 4°C and postfixed in 1% osmium tetroxide for one hour. The samples were dehydrated in increasing concentrations of ethanol (from 50%, 70%, 90% to 100%) and were critical-point-dried. Cover slips were mounted on aluminium stubs before being sputter coated with a thin layer of gold in a SC500, Bio-Rad sputter coater (Bio-Rad, BioRad Laboratories Pty Ltd, New south wales, Australia) before examination using a FEI Quanta 200 scanning electron microscope (FEI, Hillsboro, OR, USA). Backscatter imaging on *in vivo* samples was performed as described previously [6].

### Immunoflouresence

To examine focal adhesion formation and cytoskeletal organisation, osteocytes cultured on normal and OA ECM substrates were fixed in 4% paraformaldehyde (pH 7.4 in PBS) for 10 minutes, and then permeabilised with 0.2% Triton X-100 for 10 minutes. After washing and blocking with 1% BSA, cells were incubated with primary antibody vinculin 5 μg/ml (Sigma Aldrich, New south wales, Australia), SOST 5 μg/ml (R&D Systems, Sapphire Biosciences Pty Ltd, New south wales, Australia), DMP1 5 μg/ml (gift from Professor Jian Feng) and E11 5 μg/ml at 4°C overnight followed by secondary antibody (Alexa Fluor 488-labeled goat anti mouse immunoglobulin G (IgG), Invitrogen) incubation. F-actin distribution was visualised using Alexa Fluor 568 nm-labeled phalloidin (Invitrogen) staining. The samples were washed with 1X PBS three times, blown dry with air and mounted with Prolong Gold antifade solution containing DAPI for cellular nuclei staining and fluorescence preservation. Samples were then visualised using a DeltaVision PDV microscopy system equipped with an Olympus IX70 inverted microscope (Olympus). After acquisition of the *Z*-series images, they were deconvolved using the software attached in the system (Softworx; Applied Precision). The shapes of the fluorescent signals in osteocytes cultured on the normal and OA matrix were analysed using the integrated morphometry shape factor analysis of Metamorph software version 7.1.2 (Universal Imaging). This algorithm assigns a value from 0 to 1, describing the shape of the fluorescence signal where a perfect circle attains a value of 1 and a line is assigned a 0.

### Cell attachment assays

MLOY4 cells were seeded directly on normal and OA matrices with a seeding density of 10,000 viable cells per well. Cells were allowed to attach in the humidified incubator for two different time periods, 3 and 24 hours. At the end of each culture period cells were stained for 10 minutes with 0.2% crystal violet (Sigma) in 20% methanol. After washing with H_2_O, plates were dried overnight at room temperature. Cells were dissolved in 1% SDS (150 μl), and the absorbance at 570 nm was measured.

### Cell proliferation assays

Cell proliferation was determined using the CyQUANT NF Cell Proliferation Assay Kit (Molecular Probes, Invitrogen) at 48 hours of MLOY4 cell culture on normal and OA matrices according to the manufacturer’s instructions. Plates were then analysed by using a microplate reader (excitation: 485 nm, emission: 520 nm).

### Apoptosis assays

The viability of MLOY4 cells was quantified by flow cytometry with the AnnexinV-FLOUOS apoptosis staining kit (Roche Applied Science, Germany, Roche Australia PVT LTD, New south wales, Australia) following the manufacturer’s protocol.

### Quantitative real time PCR (qRT-PCR), zymography, and western blotting

qPCR, zymography and western blotting techniques were performed as described in our previously published studies [12-14,20].

### Statistical analysis

Comparisons between groups were carried out by Student’s *t*-test and for multiple comparisons analysis of variance (ANOVA) was used where *P* ≤0.05 was considered significant. Results are presented as a mean ± standard deviation (SD).

## Results

### Confirmation of de-celluarisation

**Figure 1:**
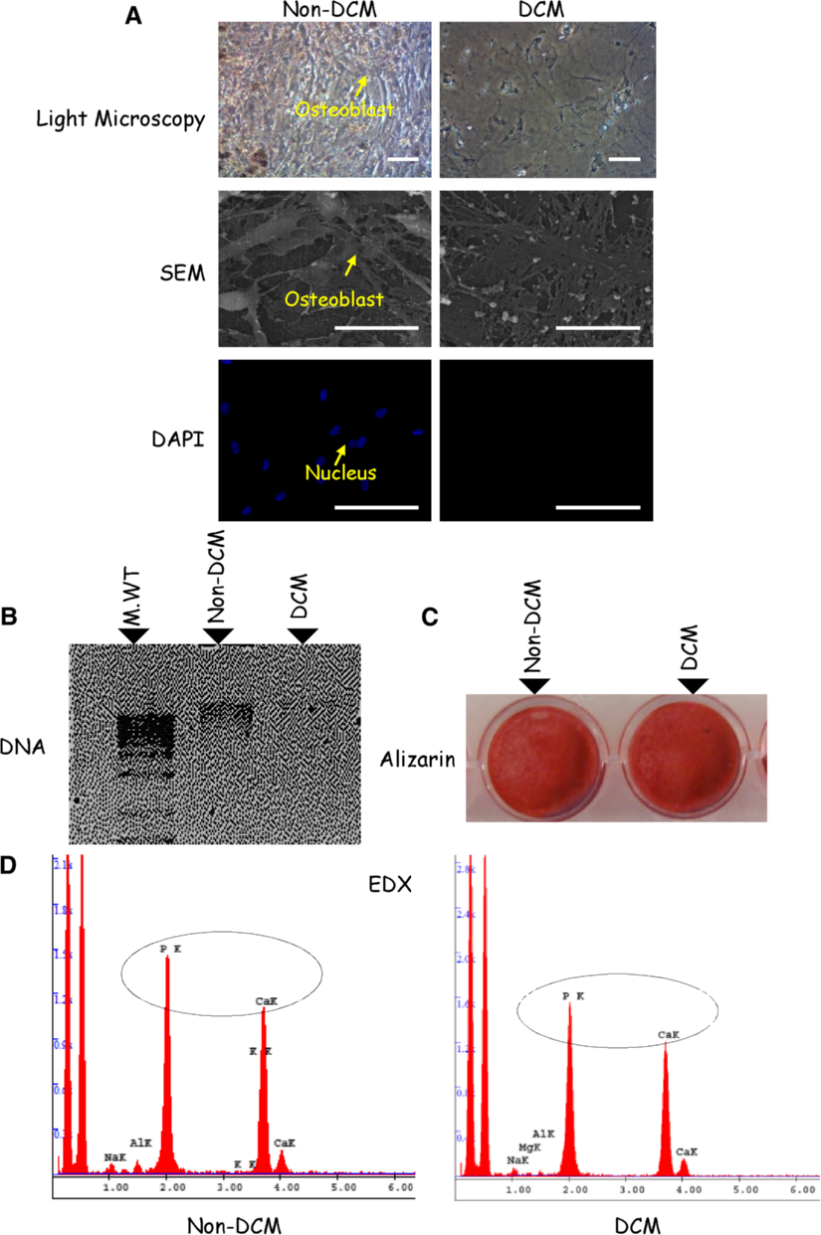
**Confirmation of de-cellularisation in osteoblast secreted matrices. (A)** Light microscopy (scale bar = 100 μm) and SEM techniques were performed following the de-celluarisation of normal matrices. Non de-celluarised osteoblasts are shown as controls. (scale bar: 20 μm). DAPI staining of de-cellularised matrices show no nuclear remnants and non de-cellularised matrices show intact nuclei (scale bar: 50 μm). **(B)** DNA analysis of the normal osteoblast derived matrices confirmed the removal of nucleic acids in the lysates of de-cellularised matrices. **(C, D)** Alizarin red staining and the EDX technique were performed to see the mineral content of the normal and OA de-cellularised matrices. All results (from A to D) are representative of matrices produced from four different patients each time with two replicates. Non DCM: Non de-cellularised matrix; DCM: de-cellularised matrix. DAPI, 4',6-diamidino-2-phenylindole; EDX, energy dispersion X-ray; OA, osteoarthritis; SEM, scanning electron microssopy.

Light microscopy and SEM analysis of de-cellularised normal matrices confirmed the intact architecture and absence of cells following the processing. Nuclear remnants could not be detected by DAPI staining, indicating satisfactory de-cellularisation of osteoblasts leaving the intact matrices. Non de-cellularised matrices were observed in parallel, showing the cell nuclear staining (Figure 1A). The absence of DNA in the matrix lysates further confirmed that approximately 99% of the DNA was removed by the de-cellularisation process (Figure 1B). Alizarin red staining confirmed the presence of mineralisation nodules after de-cellularisation (Figure 1C). Furthermore, EDX showed clear peaks for both phosphorus and calcium confirming the presence of calcium phosphate in the de-cellularised matrices obtained from the osteogenic culture of SBOs (Figure 1D). Hence, based on these results, we determined that the de-cellularisation protocol produced an acellular matrix scaffold that retains the gross, microstructural, and ultrastructural properties of the native bone tissue ECM microenvironment.

### Characterisation of ECM secreted by normal and OA osteoblasts

**Figure 2:**
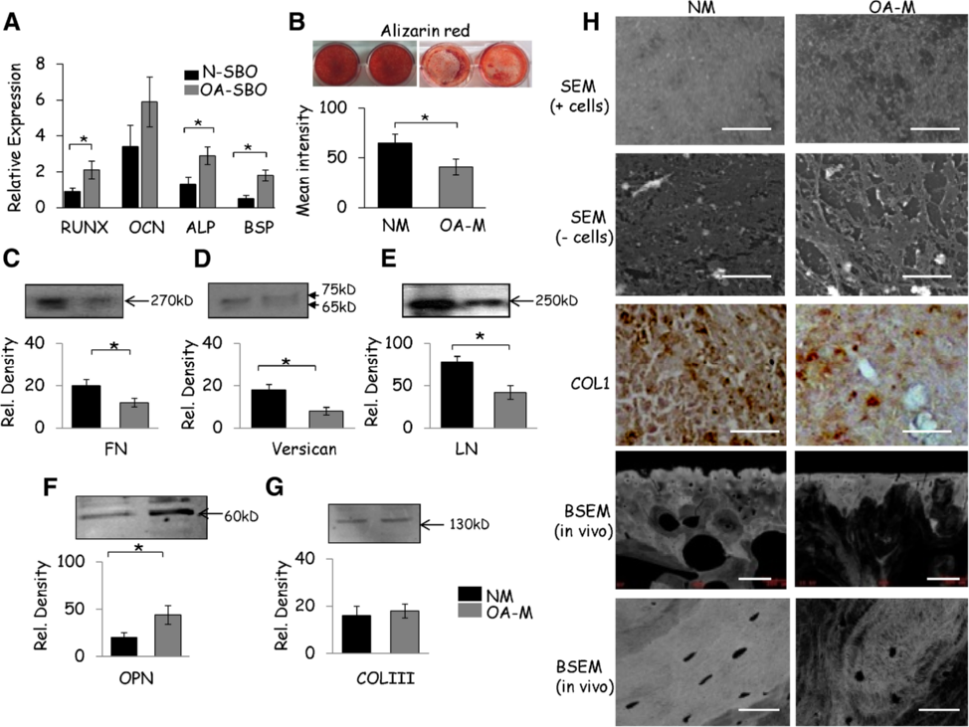
**Distribution of matrix secreted by normal and OA osteoblasts. (A)** mRNA levels of bone regulating genes in primary normal and OA subchondral bone osteoblasts (SBOs) at passage one. Values were normalised to 18srRNA and GAPDH mRNA levels (n = 5 subjects). **(B)** Normal and OA SBOs were cultured in osteogenic differentiation medium. After de-cellularisation matrix mineralisation was determined by Alizarin red. Images were taken at 20X magnification. Total staining density of each well was quantified using ImageJ software (n = 3 subjects). **(C to G)** Immunoblotting of FN, versican, LN, OPN and COLIII, in normal (NM) and OA osteoblast derived matrices (OA-M). Band density was quantified using Image J software (n = 3 subjects). **(H)** SEM micrographs and COL1 immunostaining showing the morphology of matrix organisation secreted by normal and OA osteoblasts. Note the poor alignment of OA osteoblast secreted matrix (n = 3 subjects). Scale bar:100 μm. *In vivo* evidence of disrupted bone matrix in OA patients as determined by the back scatter SEM (BS-SEM) image technique. Scale bar: 200 μm and 100 μm. Results are shown as mean ± SD, * *P* ≤0.05 (Student *t*-test).

First, at the gene level, bone regulating genes such as runt-related transcription factor (*RUNX2*), osteocalcin (*OCN*), alkaline phosphatase (*ALP*) and bone sialoprotein (*BSP*) were up-regulated in OA osteoblasts compared with normal osteoblasts (Figure 2A). Furthermore, matrix mineralisation of normal osteoblasts was higher compared to OA osteoblasts (Figure 2B). Immunoblotting was performed on the normal and OA derived de-cellularised matrices to address changes in ECM constitution specifically focussing on bone matrix RGD glycoproteins (which specifically recognise integrins). The expression of fibronectin (FN), versican and laminin (LN) was significantly higher in normal osteoblast derived matrices compared to OA osteoblast derived matrices. Surprisingly, the largest amounts of osteopontin (OPN) were present in OA osteoblast derived matrices compared to normal. COLIII was detectable in both normal and OA matrices; however this molecule did not show a significant difference (Figure 2C to G). These results demonstrate that the ECM derived from normal and OA osteoblasts were different with respect to its composition. After five weeks of osteogenic differentiation, SEM and type I collagen (COL1) immunostaining analysis demonstrate that the de-cellularised matrix formed by the OA osteoblasts had a less preserved matrix organisation and alignment compared to normal matrix. In accordance with the results obtained from the *in vitro* studies, matrix mineral distribution was disorganised, showing a woven appearance in tibial severe OA subchondral bone derived from patients undergoing knee replacement surgery (Figure 2H).

### Differential cell-matrix adhesions regulate morphology of osteocytic cells scattered within the normal and osteoarthritis osteoblast derived extracellular matrix

**Figure 3:**
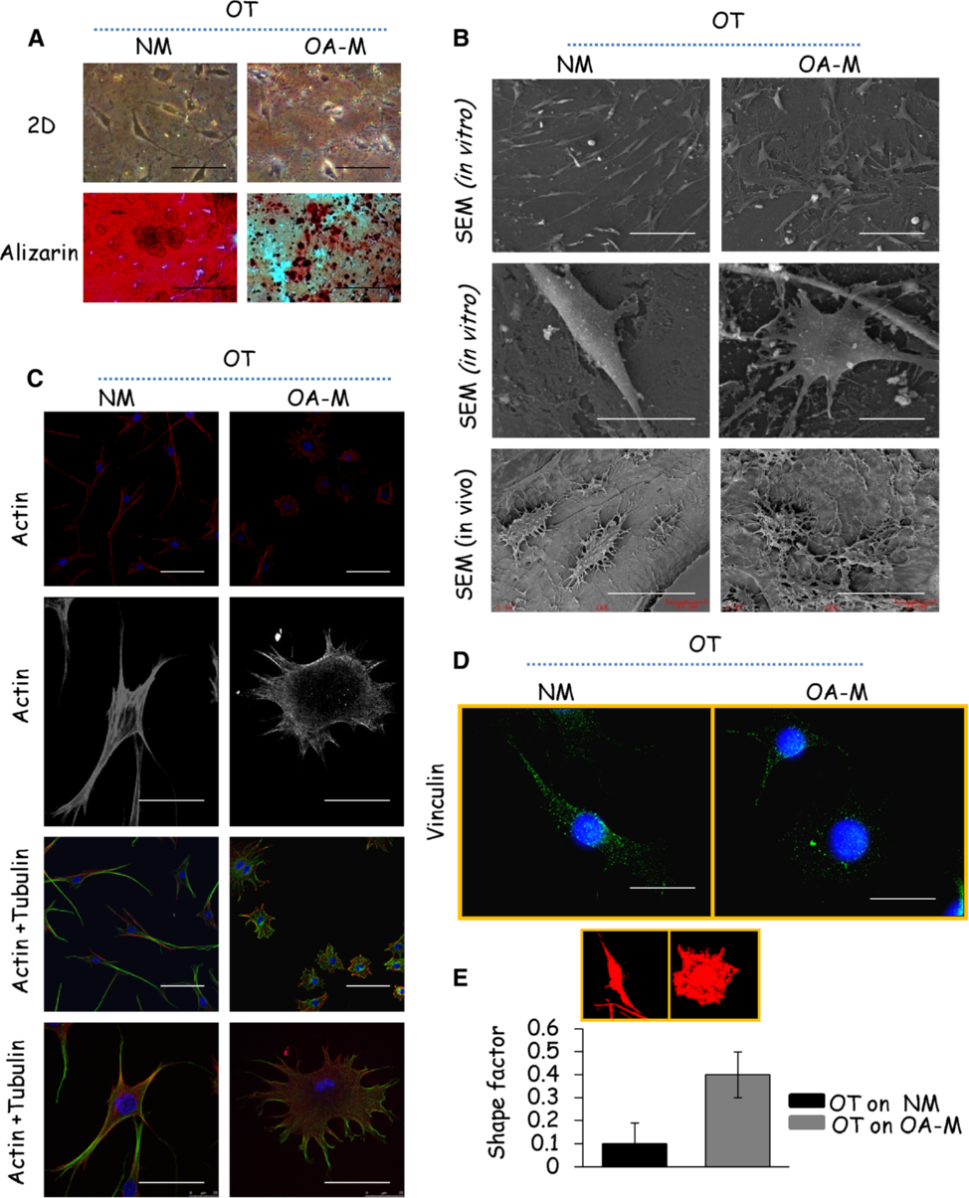
**Morphological changes of osteocytic cells in response to normal and OA osteoblast-derived matrices. (A)** Cells cultured in 2D showed rounded osteocyte morphology when grown on the OA matrices. Alizarin red combined with the haematoxylin staining showed a well aligned osteocytic cell distribution within normal matrix (NM) compared to OA matrix (OA-M). Magnification: 20X. **(B)** SEM (Scale bar: 100 μm for higher and 20 μm for lower magnification) and **(C)** confocal images (Scale bar: 50 μm for higher and 25 μm for lower magnification) indicated diversified morphologies of osteocytic cells grown on OA osteoblast derived matrices. Actin filaments are shown in red, microtubules in green and the nuclei in blue. **(D)** Representative micrographs of vinculin immunofluorescence (green) staining in osteocytic cells grown on normal and OA matrices. Scale bars: 25 μm. **(E)** Shape factor analysis of the confocal images (phalloidin only) was performed with the Metamorph software. At least 15 cells from three different experiments were analyzed. All images and graphs **(A-E)** are representative of experiments performed on osteocytic cells that were grown on four different normal and OA patient-derived matrices. Results are shown as mean ± SD. Student’s *t* test, *P* <0.05.

The morphology of osteocytic cells cultured on normal and OA osteoblast derived ECMs was examined *via* light microscopy, SEM analysis and confocal microscopy. First, basic microscopic observations of osteocytic cell populations revealed striking morphological differences when grown on normal and OA matrices. Osteocytic cells that were cultured on OA matrix typically displayed a round morphology, tended to have less protrusive extensions and their cytoplasm was wider. On the other hand, osteocytic cells that were gown on normal matrix exhibited a narrow and elongated dendritic morphology. Similarly, Alizarin red combined with haematoxylin staining revealed well organised osteocytic cells in the normal bone matrix compared to OA (Figure 3A). Based on these preliminary differences, SEM was used to further confirm these changes. SEM microphotographs showed that the osteocytic cells grown on the normal matrix revealed a dense network of dendrites, adhesion to the matrix surface and interaction between cells, whereas the cells that were grown on the OA matrices revealed round, rough and lysed morphology with poor dendrite formation and matrix alignment. We next examined the *in vivo* morphology of osteocytes in the OA subchondral bone derived from knee replacement surgeries. SEM revealed that the osteocytes in OA samples were markedly deformed with a rough, lysed and rounded appearance with very few dendrites compared to spindle shaped, well organised and well connected osteocytes in the relatively normal specimens, closely resembling the results obtained from the *in vitro* cultures as shown above. These results suggest that we could closely mimic the *in vivo* osteocyte and matrix interactions using the de-cellularised matrices (Figure 3B).

These differences in morphology suggest underlying differences in the organisation of the respective cytoskeletons. Osteocytic cells that were grown on normal matrix had straight actin fibres, which were visualised by phalloidin. When the cells were grown on the OA matrix, actin filament fibres were observed randomly and visually no fibres were present at the center of the osteocytic cells (Figure 3C).

The formation of focal adhesion complexes is a prerequisite for cell-ECM adhesion. Therefore, it is possible that the altered cell morphology capabilities of osteocytic cells on the OA matrix may result from alterations in focal adhesion functions. We thus used immunofluorescence staining to examine the expression of focal adhesions. As shown in Figure 3D, osteocytic cells grown on normal matrices formed multiple focal adhesion structures at plasma membranes and cytoplasm, as visualised by staining for vinculin, an abundant cytoskeleton protein localised at focal adhesions. In marked contrast, osteocytic cells on an OA matrix showed fewer focal adhesion contact points. Confocal images of osteocytic cells shown in Figure 3C were quantitatively analysed for shape factor. Cell shape factor changed from 0.1 to 0.4, indicating a transformation from a non-circular spreading pattern as the cells developed spindle shaped morphology to a rounded/circle pattern when grown on an OA matrix (Figure 3E).

### Altered functional and gene expression characteristics of osteocytic cells grown on osteoarthritis osteoblast-derived matrices

**Figure 4:**
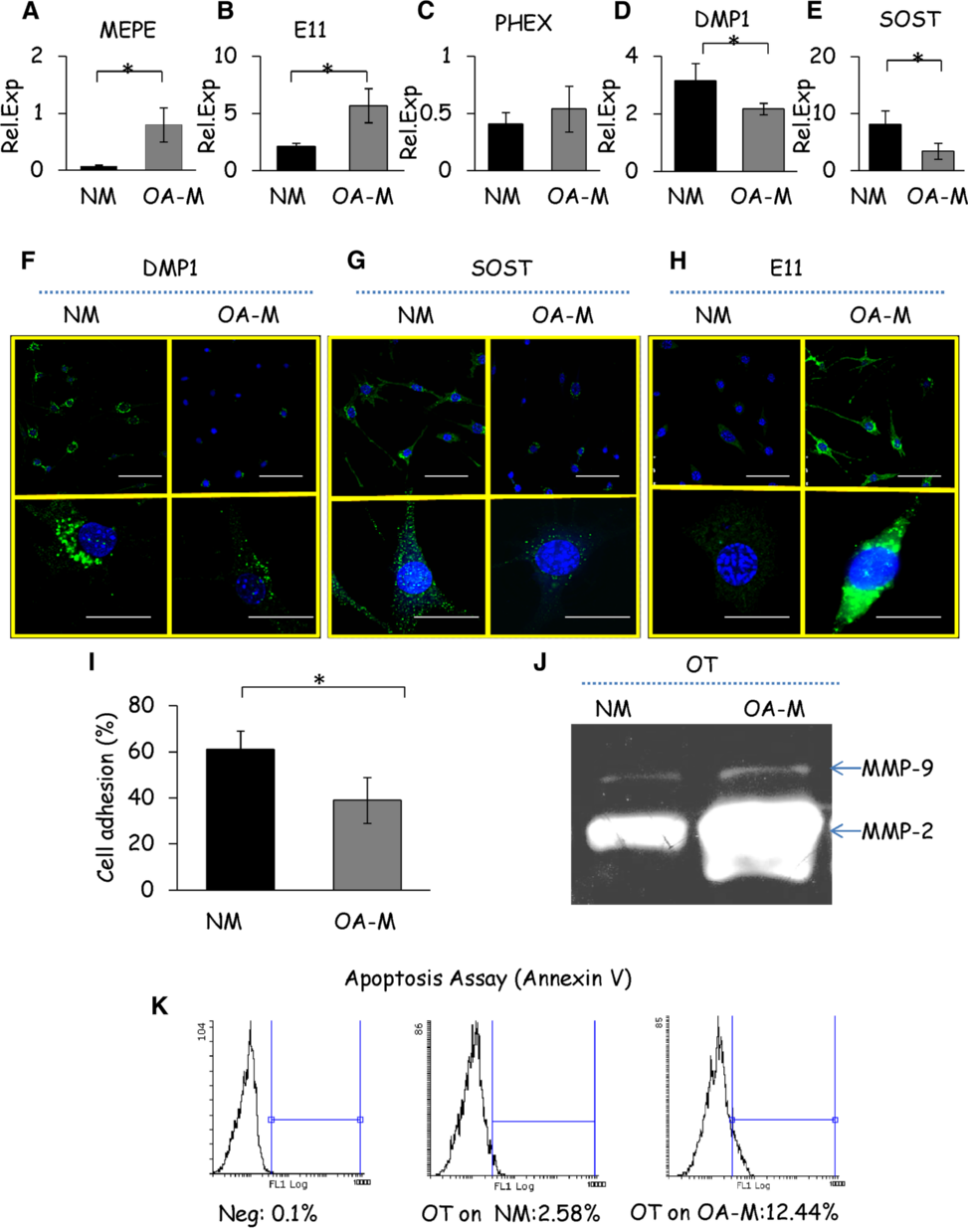
**Functional and gene associated changes of osteocytic cells (OT) in response to normal (NM) and OA osteoblast derived matrices (OA-M). (A to E)** Gene expression profiles of osteocyte markers were determined by qRT-PCR in osteocytic cells cultured either on normal or OA matrices for 48 hours. The results are expressed as relative gene expression after normalization using the 18srRNA and GAPDH housekeeping gene. **(F to H)** Protein localisation of DMP1, SOST and E11 osteocyte markers was determined by confocal microscopy (Scale bar: 50 μm for higher and 25 μm for lower magnification). **(I)** Cell adhesion of osteocytic cells cultured on normal and OA matrices was determined by staining with crystal violet. **(J)** Zymographic analysis shows the expression of MMP2 and MMP9 in osteocyte conditioned media collected after 24 hours of incubation after culturing on normal and OA matrices. **(K)** The flow cytometric apoptosis analysis of osteocytic cells on normal and OA matrices was determined by Annexin V/PI staining. All images and graphs are representative of experiments performed on osteocytic cells cultured on matrices derived from four different normal and OA patients. Results are shown as mean ± SD, * *P* ≤0.05 by Student’s *t*-test.

To understand the regulation of osteocyte function on the normal and OA matrices, we compared osteocyte specific gene expression, cell attachment, apoptosis and Matrix metalloproteinases (MMP-2) and -9 production. Using real-time PCR, we analysed a panel of characteristic osteocyte markers after 48 hours of osteocytic cell culture on normal and OA matrices. We observed that the expression of matrix extracellular phosphoglycoprotein (MEPE) and E11 were upregulated significantly when osteocytic cells were grown on OA matrices. On the other hand, the expression of phosphate-regulating gene with homology to endopeptidases on X chromosome (PHEX) remained at similar levels with no differences between normal and OA matrices. In contrast, DMP1 and SOST were significantly downregulated in osteocytic cells that were cultured on the OA matrices compared to those that were grown on normal matrix. (Figure 4A to E). Of note, although the MLOY4 cell line is a very powerful tool for the study of osteocytes *in vitro*[21-23], there are known differences between primary osteocytes and the immortalised MLOY4 cell line. For example, MLOY4 cells express low levels of DMP1 and SOST, while osteocytes are known to express these genes *in vivo*[24,25]. In this study, we observed that MLOY4 cells cultured on the collagen I coated flasks expressed relatively low levels of dentin matrix protein 1 (DMP1) and sclerostin (SOST) (data not shown); however, when these cells were grown on the normal and OA matrices we found a significant increase in the expression of these proteins compared to those grown on the COL1 coated flasks. These results suggest that the embedding of MLOY4 cells in the mineralised matrices may significantly enhance the expression of these mature osteocyte markers, closely mimicking the native bone matrix osteocyte phenotype. Confocal microscopy imaging of the DMP1, SOST and E11 demonstrated trends similar to those seen at the gene expression (Figure 4F, G,H) level. When the ability of cell attachment was compared between normal and OA matrices, at three hours osteocytic cells on normal and OA matrices had settled onto their respective substrates and had a rounded morphology (data not shown). However, at 24 hours the osteocytic cells were attached more on normal matrix compared to those that were grown on the OA matrix as determined by the crystal violet adhesion assay (Figure 4I). Because we observed a lysed morphology of osteocytic cells on the OA matrix, we compared the expression of MMP-2 and MMP-9 by zymography as these molecules are well known to be involved in cell–matrix adhesions. We found higher levels of MMP-2 and MMP-9 in cells that were cultured on the OA matrices (Figure 4J). Furthermore, osteocytic cells exposed to OA bone matrix appeared to be more sensitive to apoptosis than those cultured on the normal matrices (Figure 4K).

### Expression of integrinβ1-FAK signalling in osteocytic cells cultured in normal and osteoarthritis bone matrices

**Figure 5:**
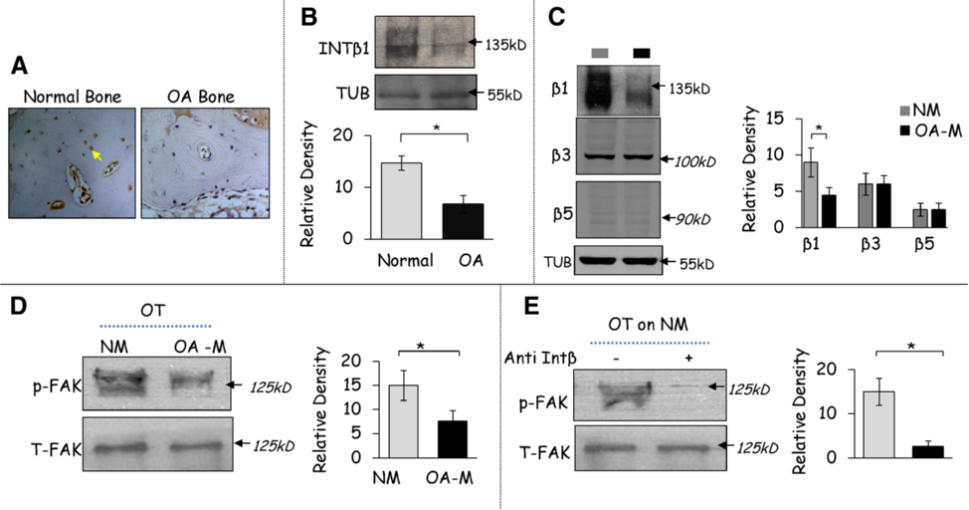
**Expression of integrinβ1(INT-β1) - FAK signalling in osteocytic cells grown on normal and OA matrix. (A, B)** Immunohistochemical and western blot analysis shows a decrease in the positively stained osteocytes (OT) for integrinβ1 in OA subchondral bone compared to normal bone collected from the human tibia. Images are representative of the experiments performed on three different patients. **(C, D)** Western blot analysis of integrinβ1, αVβ5 and αVβ3 and FAK phosphorylation in osteocytic cells that were grown on OA matrices (OA-M) compared to normal matrices (NM). Band density graphs are representative of the experiments performed on three different patient matrices. Results are shown as mean ± SD, * *P* ≤0.05 by Student’s *t*-test. **(E)** Decreased phosphorylation of FAK upon blocking integrinβ1 in osteocytic cells cultured on the normal matrices (NM). Images and graphs are representative of experiments performed on three patient matrices developed from normal and OA osteoblasts. Results are shown as mean ± SD, * *P* ≤0.05 by Student’s *t*-test.

In immunohistochemistry analysis, *in vivo* osteocytes in relatively normal human subchondral bone expressed higher levels of integrinβ1 compared to osteocytes from OA patients, indicating the altered expression of this molecule in OA osteocytes. This downregulation of integrinβ1 protein expression in human OA bone tissue lysate was further confirmed by immunoblotting (Figure 5A,B). Furthermore, the expression of integrinβ1 was significantly higher in osteocytic cells that were grown on the normal matrices compared to OA matrices (OA-M). On the other hand, the expression levels of αVβ5 and αVβ3 integrins that also recognise RGD remained unchanged (Figure 5C). Integrinβ1 can serve as an important regulator of downstream signalling. We, therefore, examined modifications of the intracellular signalling cascade in osteocytic cells after exposure to normal and OA matrices. The experiments concentrated on FAK signalling pathways which are known to be involved in cell-matrix regulation. Also, compared to the OA matrices the osteocytic cells cultured on normal matrices evoked upregulation of FAK phosphorylation (Figure 5D). Furthermore, integrinβ1 inhibition using blocking antibodies led to FAK dephosphorylation even when osteocytic cells were cultured on normal matrices indicating that the phosphorylation of FAK was dependent on integrinβ1 activation (Figure 5E).

### Integrinβ1 blocking diminished phenotypic and genotypic capacity of osteocytic cells attached on the normal matrices

**Figure 6:**
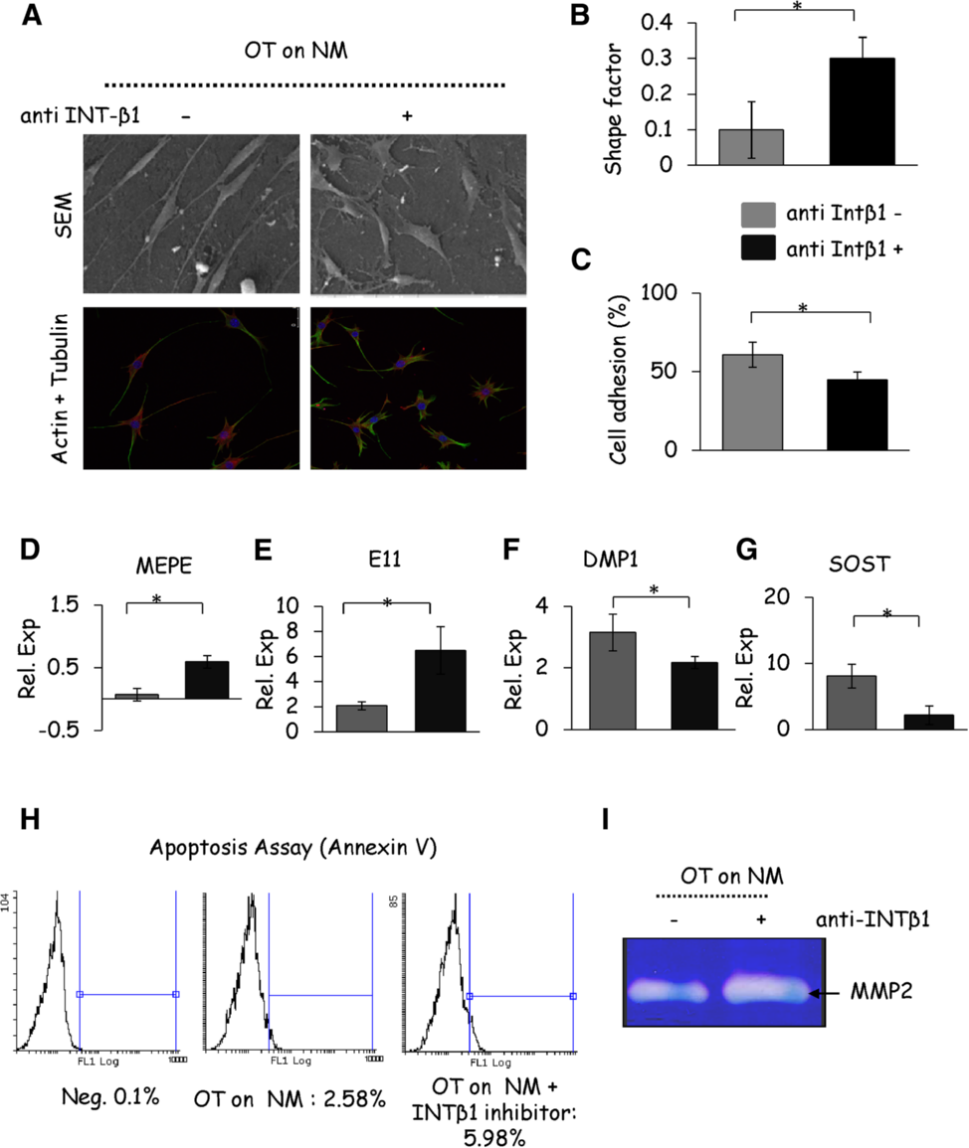
**Integrinβ1 (INT-β1) blocking disables the morphology and function of osteocytic cells cultured on normal matrices. (A)** SEM and confocal images illustrating distinct morphological changes induced by integrinβ1 blocking in osteocytic cells cultured on the normal matrices (NM). (Scale bars: 50 μm). **(B)** Shape factor determined on the osteocytic cells cultured on normal matrices upon integrinβ1 blocking. **(C)** Cell adhesion determined on the osteocytic cells grown on the normal matrices upon integrinβ1 blocking **(D to G)** Gene expression profiles of osteocyte markers as determined by qRT-PCR in the presence of integrinβ1 blocking antibodies on normal matrix. The results are expressed as relative gene expression after normalisation using the 18srRNA and GAPDH housekeeping gene. **(H)** Osteocytic cells grown on the normal matrices were treated with integrinβ1 blocking antibody and incubated for 24 hours, and then detection of apoptosis was performed by flow cytometry. **(I)** Zymographic analysis shows the expression of MMP2 in osteocytic cell conditioned media collected after 24 hours of incubation after culturing on normal matrices in the presence or absence of integrinβ1 blocking antibody. All images and graphs are repremsentative of experiments performed on osteocytic cells cultured on matrices derived from three different normal patients. Results are shown as mean ± SD, * *P* ≤0.05 by Student’s *t*-test.

Next we examined the relevance of integrinβ1 in mediating the phenotypic and genotypic responses of osteocytic cells cultured on the normal matrices by using blocking antibodies. In this context, as determined by SEM and confocal microscopy, the morphology of osteocytic cells grown on normal matrices in the presence of integrinβ1 blocking antibodies was drastically changed to a phenotype similar to cells that were grown on the OA matrices, demonstrating a link between integrinβ1 and altered osteocyte cell-matrix interaction (Figure 6A,B). Furthermore, osteocytic cell adhesion was decreased in the presence of integrinβ1 blocking antibodies (Figure 6C). In addition, the osteocyte markers E11 and MEPE were upregulated and genes, such as *DMP1* and *SOST*, were significantly downregulated upon blocking of integrinβ1 (Figure 6D to G). Likewise, blocking antibodies against integrinβ1 increased osteocyte apoptosis (Figure 6H) and MMP production (Figure 6I). These results together suggest an important role of integrinβ1 in mediating the osteocyte and cell matrix interactions under normal conditions.

## Discussion

It is now well accepted that joint cartilage degeneration is associated with intensified remodelling of the subchondral bone and increased bone stiffness [26]. Understanding which cells/molecules are involved in bone sclerosis could help us find ways to manipulate such molecules to slow down the progression of OA.

Earlier studies have shown that the de-cellularised matrix produced by osteoblasts is able to induce attachment and spreading of many adherent cell types, including mesenchymal stem cells [27-29]. In principle, the use of NH4OH leads to hypoxia and subsequent cell lysis leaving the matrix intact. It is thus regarded as an *in vitro* model to study the molecular basis of cell attachment, spreading and signalling from the ECM. In the present study, we confirmed that de-cellularised osteoblast matrices represent the *in vivo* bone matrix characterised by accumulation of the mineral content and expression of various collagenous and non-collagenous proteins, thus providing the ideal environment to study cell-matrix interactions. First, we observed significant differences in the mineral content of matrices secreted by normal and OA osteoblasts. These results are in agreement with previous reports suggesting the abnormally low mineralisation of OA osteoblasts [30]. The present study also showed that human osteoblast ECM derived from primary bone cell cultures has the capacity to produce a wide range of matrix proteins. Surprisingly, we observed that the levels of fibronectin, laminin and versican were decreased in OA matrices compared to normal. In general, fibronectin is presumed to contribute more to general structure and load bearing [31], whereas versican and laminin interact with other matrix components as well as with cell surface adhesion receptors via well-defined domains [32]. Low expression of these molecules is suggestive of abnormal ECM composition in OA matrices. Previous studies demonstrated an increase of COL1 in OA osteoblasts at the cellular level [33]. In this study, the matrix deposited by OA osteoblasts showed a dispersed COL1 staining compared to normal matrix. These results corroborate the results of studies showing disorganised collagen fibres in OA bone compared to normal [34]. Furthermore, although not statistically significant, we observed that expression of OCN was higher in OA patients. However, many other studies showed a significant increase of OCN in OA patients [30,35]. This disparity in the results could be attributed to biological variation, process variation and system variation.

Based on the above results, it is apparent that the ECM secreted by normal and OA osteoblasts is different. We next tested whether these matrix differences can contribute to the poor morphological and phenotypic properties of osteocytes seen in OA bone. Our results suggest that osteocyte cell lines, such as MLOY4, could be altered in response to the ECM of OA osteoblasts compared to the ECM generated from normal osteoblasts. At the gene expression level, when osteocytic cells were grown on OA matrices a decrease in DMP1 and SOST expression and an increase in E11 and MEPE expression were found. Although E11, MEPE, DMP1 and SOST are known markers of the osteocyte, each gene has a distinct function and their expression levels change according to the specific stage of differentiation. Osteocyte expression of SOST [36] and DMP [37] is a delayed event and it is produced only by osteocytes after they become embedded deeply in matrix that has been fully mineralised. Our results showed that OA osteoblasts failed to lay a proper mineral or matrix. Therefore, these matrix properties might have influenced the behaviour of osteocytes, stopping them from expressing those mature markers; whereas E11 and MEPE are early markers and they start to express when osteocytes are in the process of embedding into the matrix that is not fully mineralised [38]. Failure of osteocytic cells to produce these proteins when cultured on the OA matrix also suggests a state of immature osteocytes. These results together suggest that in OA matrices osteocytic cells remained in an immature phenotype; however, when they were cultured on the normal matrices, the osteocytes differentiated from an immature to mature phenotype. Likewise, on the OA matrices an increase in osteocytic cell death was observed which can possibly be related to increased skeletal fragility, linked to the loss of ability to sense microdamage and/or signal repair of *in vivo* bone [39].

The observation that the morphological and phenotypic characteristics of osteocytic cells attached to the normal and the OA matrices differ from each other, suggests that specific signalling pathways must arise or alter between matrix and cells. Of note, we observed that the expression of integrinβ1 was much less in osteocytic cells that were cultured on the OA matrices compared to normal matrices indicating that the decrease of integrinβ1 expression could be responsible for the observed phenotypic changes of osteocytes in OA.

How and to what extent integrinβ1 is involved in the transduction of a matrix signal to modulate osteocyte function was investigated by a series of further experiments. We found that the blocking integrinβ1 activity promoted the elongated cell morphology to a rounded phenotype even when osteocytic cells are cultured on normal osteoblast matrices. This series of events occurred *via* the down regulation of FAK phosphorylation levels. The effect of integrinβ1 blocking on cell morphology was possibly due to FAK signals leading to actin contractility events and the dynamic regulation of viniculin. Normally, integrinβ1-FAK signalling, apart from playing a role as a hook to attach the cell to matrix, generates a signal to enforce the cell towards functions such as attachment, apoptosis and differentiation. In this study, we observed that osteocytic cells on OA matrices and integrinβ1 blocking showed an increased apoptosis and altered osteocyte gene expression, indicating that the expression of integrinβ1-FAK signalling is important to maintain the normal osteocyte-matrix interactions. *In vitro* data presented here are consistent with results from an integrinβ1 negative mouse model in which mice exhibited less mineralised bone, reduced tibial curvature and decreased femoral strength [40]. Thus, this study provides evidence that integrinβ1 may initiate intracellular signals either by organisation of the cytoskeleton and alteration of cell shape or through mechanisms akin to osteocyte signalling. In this study, we observed that the expression of Vβ3 and Vβ5 were not changed. Although different integrin receptors perform common functions and can share identical ligands, each member seems to be highly specific, since mice carrying gene deletions of the different integrin chains often show non-overlapping phenotypes [41]. We are confident that there might be other integrins participating in the interaction and further studies are warranted to determine their role.

## Conclusion

In summary, this study demonstrated that ECM from OA osteoblasts can induce significant alteration of osteocytic cells via a focal adhesion mediated integrinβ1-FAK cell signalling pathway, a possible mechanism of OA subchondral bone sclerosis and OA progression.

## Abbreviations

ALP: Alkaline phosphatase; BSA: Bovine serum albumin; BSEM: Back scattered electron microscopy; BSP: Bone sialo protein; COL1: Type I collagen; COLIII: Type III collagen; DAPI: 4',6-diamidino-2-phenylindole; DCM: De-cellularised matrix; DMP: Dentin matrix protein; DMP1: Dentin matrix protein 1; (D)MEM: (Dulbecco’s)_modified Eagle’s medium; ECM: Extracellular matrix; EDX: Energy dispersive X-ray; FAK: Focal adhesion kinase; FN: Fibronectin; INTb1: Integrin beta1; LN: Laminin; MEPE: Matrix extracellular phosphoglycoprotein; MMP: Matrix metalloproteinases; OA: Osteoarthritis matrix; OCN: osteocalcin; OPN: Osteopontin; OT: Osteocytes; PBS: Phosphate-buffered saline; PHEX: Phosphate-regulating neutral endopeptidase; qPCR: Quantitative polymerase chain reaction; SBO: Subchondral bone osteoblasts; SEM: Scanning electron microscopy; SOST: Sclerostin; TUB: Tubulin.

## Competing interests

The authors declare that they have no competing interests.

## Authors’ contributions

IP planned the studies, performed experiments, analysed data and wrote the manuscript. SF, WG, SP, JF and RC did experiments and analysed data. YX supervised the project, planned studies, analysed data and wrote the manuscript. All authors have been involved in drafting the manuscript or in revising it critically for important intellectual content and have read and approved the final manuscript.

## References

[B1] Green J Schotland S Stauber DJ Kleeman CR Clemens TL Cell-matrix interaction in bone: type I collagen modulates signal transduction in osteoblast-like cells Am J Physiol 1995 15 C1090 C1103 7762601 10.1152/ajpcell.1995.268.5.C1090

[B2] Popov C Radic T Haasters F Prall WC Aszodi A Gullberg D Schieker M Docheva D Integrins alpha2beta1 and alpha11beta1 regulate the survival of mesenchymal stem cells on collagen I Cell Death Dis 2011 15 e186 10.1038/cddis.2011.71 21796158 PMC3199721

[B3] Zohar R Tal PH Signals between cells and matrix mediate bone regeneration Bone Regeneration 2012 InTech Available from: http://www.intechopen.com/books/bone-regeneration/signals-between-cells-and-matrix-mediate-bone-regeneration

[B4] Burr DB The importance of subchondral bone in the progression of osteoarthritis J Rheumatol Suppl 2004 15 77 80 15132360

[B5] Neve A Corrado A Cantatore FP Osteocytes: central conductors of bone biology in normal and pathological conditions Acta Physiol (Oxf) 2012 15 317 330 10.1111/j.1748-1716.2011.02385.x 22099166

[B6] Jaiprakash A Prasadam I Feng JQ Liu Y Crawford R Xiao Y Phenotypic characterization of osteoarthritic osteocytes from the sclerotic zones: a possible pathological role in subchondral bone sclerosis Int J Biol Sci 2012 15 406 417 22419886 10.7150/ijbs.4221 PMC3303142

[B7] Lu XL Huo B Chiang V Guo XE Osteocytic network is more responsive in calcium signaling than osteoblastic network under fluid flow J Bone Miner Res 2012 15 563 574 10.1002/jbmr.1474 22113822 PMC3343217

[B8] Bonewald LF Osteocyte biology: its implications for osteoporosis J Musculoskelet Neuronal Interact 2004 15 101 104 15615083

[B9] Schaffner P Dard MM Structure and function of RGD peptides involved in bone biology Cell Mol Life Sci 2003 15 119 132 10.1007/s000180300008 12613662 PMC11138839

[B10] Robey PG Fedarko NS Hefferan TE Bianco P Vetter UK Grzesik W Friedenstein A Van der Pluijm G Mintz KP Young MF Structure and molecular regulation of bone matrix proteins J Bone Miner Res 1993 15 S483 S487 8122516 10.1002/jbmr.5650081310

[B11] Legate KR Wickstrom SA Fassler R Genetic and cell biological analysis of integrin outside-in signaling Genes Dev 2009 15 397 418 10.1101/gad.1758709 19240129

[B12] Prasadam I Crawford R Xiao Y Aggravation of ADAMTS and matrix metalloproteinase production and role of ERK1/2 pathway in the interaction of osteoarthritic subchondral bone osteoblasts and articular cartilage chondrocytes – possible pathogenic role in osteoarthritis J Rheumatol 2012 15 621 634 10.3899/jrheum.110777 22247346

[B13] Prasadam I Friis T Shi W van Gennip S Crawford R Xiao Y Osteoarthritic cartilage chondrocytes alter subchondral bone osteoblast differentiation via MAPK signalling pathway involving ERK1/2 Bone 2010 15 226 235 10.1016/j.bone.2009.10.014 19853676

[B14] Prasadam I van Gennip S Friis T Shi W Crawford R Xiao Y ERK-1/2 and p38 in the regulation of hypertrophic changes of normal articular cartilage chondrocytes induced by osteoarthritic subchondral osteoblasts Arthritis Rheum 2010 15 1349 1360 10.1002/art.27397 20155832

[B15] Altman R Asch E Bloch D Bole G Borenstein D Brandt K Christy W Cooke TD Greenwald R Hochberg M Development of criteria for the classification and reporting of osteoarthritis. Classification of osteoarthritis of the knee. Diagnostic and Therapeutic Criteria Committee of the American Rheumatism Association Arthritis Rheum 1986 15 1039 1049 10.1002/art.1780290816 3741515

[B16] Beresford JN Gallagher JA Gowen M McGuire MKB Poser JW Russell RG Human bone cells in culture: a novel system for the investigation of bone cell metabolism Clin Sci (Colch) 1983 15 38 39

[B17] Beresford JN Gallagher JA Poser JW Russell RG Production of osteocalcin by human bone cells in vitro. Effects of 1,25(OH)2D3, 24,25(OH)2D3, parathyroid hormone, and glucocorticoids Metab Bone Dis Relat Res 1984 15 229 234 10.1016/0221-8747(84)90064-X 6333574

[B18] Kato Y Windle JJ Koop BA Mundy GR Bonewald LF Establishment of an osteocyte-like cell line, MLO-Y4 J Bone Miner Res 1997 15 2014 2023 9421234 10.1359/jbmr.1997.12.12.2014

[B19] Mao X Peng H Ling J Friis T Whittaker AK Crawford R Xiao Y Enhanced human bone marrow stromal cell affinity for modified poly(L-lactide) surfaces by the upregulation of adhesion molecular genes Biomaterials 2009 15 6903 6911 10.1016/j.biomaterials.2009.09.013 19796804

[B20] Prasadam I Mao X Wang Y Shi W Crawford R Xiao Y Inhibition of p38 pathway leads to OA-like changes in a rat animal model Rheumatology (Oxford) 2012 15 813 823 10.1093/rheumatology/ker360 22240502

[B21] Marathe N Rangaswami H Zhuang S Boss GR Pilz RB Pro-survival effects of 17beta-estradiol on osteocytes are mediated by nitric oxide/cGMP via differential actions of cGMP-dependent protein kinases I and II J Biol Chem 2012 15 978 988 10.1074/jbc.M111.294959 22117068 PMC3256896

[B22] Thompson WR Majid AS Czymmek KJ Ruff AL Garcia J Duncan RL Farach-Carson MC Association of the alpha(2)delta(1) subunit with Ca(v)3.2 enhances membrane expression and regulates mechanically induced ATP release in MLO-Y4 osteocytes J Bone Miner Res 2011 15 2125 2139 10.1002/jbmr.437 21638318 PMC4478606

[B23] Burra S Nicolella DP Francis WL Freitas CJ Mueschke NJ Poole K Jiang JX Dendritic processes of osteocytes are mechanotransducers that induce the opening of hemichannels Proc Natl Acad Sci U S A 2010 15 13648 13653 10.1073/pnas.1009382107 20643964 PMC2922284

[B24] Stern AR Stern MM Van Dyke ME Jahn K Prideaux M Bonewald LF Isolation and culture of primary osteocytes from the long bones of skeletally mature and aged mice Biotechniques 2012 15 361 373 22668415 10.2144/0000113876 PMC3612989

[B25] Paic F Igwe JC Nori R Kronenberg MS Franceschetti T Harrington P Kuo L Shin DG Rowe DW Harris SE Kalajzic I Identification of differentially expressed genes between osteoblasts and osteocytes Bone 2009 15 682 692 10.1016/j.bone.2009.06.010 19539797 PMC2731004

[B26] Lajeunesse D Reboul P Subchondral bone in osteoarthritis: a biologic link with articular cartilage leading to abnormal remodeling Curr Opin Rheumatol 2003 15 628 633 10.1097/00002281-200309000-00018 12960492

[B27] Reichert JC Quent VMC Burke LJ Stansfield SH Clements JA Hutmacher DW Mineralized human primary osteoblast matrices as a model system to analyse interactions of prostate cancer cells with the bone microenvironment Biomaterials 2010 15 7928 7936 10.1016/j.biomaterials.2010.06.055 20688384

[B28] Grunert M Dombrowski C Sadasivam M Manton K Cool SM Nurcombe V Isolation of a native osteoblast matrix with a specific affinity for BMP2 J Mol Histol 2007 15 393 404 10.1007/s10735-007-9119-0 17682830

[B29] Dumas V Ducharne B Perrier A Fournier C Guignandon A Thomas M Peyroche S Guyomar D Vico L Rattner A Extracellular matrix produced by osteoblasts cultured under low-magnitude, high-frequency stimulation is favourable to osteogenic differentiation of mesenchymal stem cells Calcif Tissue Int 2010 15 351 364 10.1007/s00223-010-9394-8 20582583

[B30] Sanchez C Deberg MA Bellahcene A Castronovo V Msika P Delcour JP Crielaard JM Henrotin YE Phenotypic characterization of osteoblasts from the sclerotic zones of osteoarthritic subchondral bone Arthritis Rheum 2008 15 442 455 10.1002/art.23159 18240211

[B31] Batra N Burra S Siller-Jackson AJ Gu S Xia X Weber GF DeSimone D Bonewald LF Lafer EM Sprague E Schwartz MA Jiang JX Mechanical stress-activated integrin alpha5beta1 induces opening of connexin 43 hemichannels Proc Natl Acad Sci USA 2012 15 3359 3364 10.1073/pnas.1115967109 22331870 PMC3295295

[B32] Nishimoto SK Nishimoto M Matrix Gla protein C-terminal region binds to vitronectin. Co-localization suggests binding occurs during tissue development Matrix Biol 2005 15 353 361 10.1016/j.matbio.2005.05.004 15982861

[B33] Couchourel D Aubry I Delalandre A Lavigne M Martel-Pelletier J Pelletier JP Lajeunesse D Altered mineralization of human osteoarthritic osteoblasts is attributable to abnormal type I collagen production Arthritis Rheum 2009 15 1438 1450 10.1002/art.24489 19404930 PMC5250342

[B34] Bailey AJ Sims TJ Knott L Phenotypic expression of osteoblast collagen in osteoarthritic bone: production of type I homotrimer Int J Biochem Cell Biol 2002 15 176 182 10.1016/S1357-2725(01)00107-8 11809420

[B35] Cantatore FP Corrado A Grano M Quarta L Colucci S Melillo N Osteocalcin synthesis by human osteoblasts from normal and osteoarthritic bone after vitamin D3 stimulation Clin Rheumatol 2004 15 490 495 10.1007/s10067-004-0928-1 15801068

[B36] Atkins GJ Rowe PS Lim HP Welldon KJ Ormsby R Wijenayaka AR Zelenchuk L Evdokiou A Findlay DM Sclerostin is a locally acting regulator of late-osteoblast/preosteocyte differentiation and regulates mineralization through a MEPE-ASARM-dependent mechanism J Bone Miner Res 2011 15 1425 1436 10.1002/jbmr.345 21312267 PMC3358926

[B37] Kalajzic I Braut A Guo D Jiang X Kronenberg MS Mina M Harris MA Harris SE Rowe DW Dentin matrix protein 1 expression during osteoblastic differentiation, generation of an osteocyte GFP-transgene Bone 2004 15 74 82 10.1016/j.bone.2004.03.006 15207743

[B38] Woo SM Rosser J Dusevich V Kalajzic I Bonewald LF Cell line IDG-SW3 replicates osteoblast-to-late-osteocyte differentiation in vitro and accelerates bone formation in vivo J Bone Miner Res 2011 15 2634 2646 10.1002/jbmr.465 21735478 PMC3192242

[B39] Qiu S Sudhaker Rao D Fyhrie DP Palnitkar S Parfitt AM The morphological association between microcracks and osteocyte lacunae in human cortical bone Bone 2005 15 10 15 10.1016/j.bone.2005.01.023 15878702

[B40] Globus RK Amblard D Nishimura Y Iwaniec UT Kim JB Almeida EA Damsky CD Wronski TJ van der Meulen MC Skeletal phenotype of growing transgenic mice that express a function-perturbing form of beta1 integrin in osteoblasts Calcif Tissue Int 2005 15 39 49 10.1007/s00223-004-0309-4 15477996

[B41] Chen C Sheppard D Identification and molecular characterization of multiple phenotypes in integrin knockout mice Methods Enzymol 2007 15 291 305 17697889 10.1016/S0076-6879(07)26013-6

